# Targeting *CD226*/DNAX accessory molecule-1 (DNAM-1) in collagen-induced arthritis mouse models

**DOI:** 10.1186/s12950-015-0056-5

**Published:** 2015-02-08

**Authors:** Muriel Elhai, Gilles Chiocchia, Carmen Marchiol, Franck Lager, Gilles Renault, Marco Colonna, Guenter Bernhardt, Yannick Allanore, Jérôme Avouac

**Affiliations:** Rheumatology A department, Cochin Hospital, Paris Descartes University, Sorbonne Paris Cité, 27 rue du Faubourg Saint Jacques, 75014 Paris, France; Cochin Institut, INSERM U1016, UMR 8104, Team ATIP/AVENIR, Paris Descartes University, Paris, France; Inserm U987, Université Versailles-Saint-Quentin, Montigny-Le-Bretonneux, France; Small Animal Imaging Facility, Paris Descartes University, INSERM U1016, Institut Cochin, Sorbonne Paris Cité, Paris, France; Department of Pathology and Immunology, Washington University School of Medicine, St Louis, USA; Institute of Immunology, Hannover Medical School, Hannover, Germany

**Keywords:** Rheumatoid arthritis, Collagen-induced arthritis, CD226, Autoimmunity, Mouse model

## Abstract

**Background:**

Genetic studies have pointed out that *CD226* variants, encoding DNAM-1, could be associated with susceptibility to rheumatoid arthritis. Therefore, we aimed to determine the influence of DNAM-1 on the development of arthritis using the collagen-induced arthritis (CIA) mouse model.

**Methods:**

CIA was induced in mice on a DBA/1 background, treated in parallel with a DNAM-1 neutralizing monoclonal antibody, a control IgG and PBS, respectively. CIA was also induced in mice deficient for DNAM-1(dnam1−/−) and control dnam-1+/+ mice on a C57/BL6 background. Mice were monitored for clinical and ultrasound signs of arthritis. Histological analysis was performed to search for inflammatory infiltrates and erosions. The Mann–Whitney U test for non-related samples was used for statistical analysis.

**Results:**

There was a non-significant trend for a less arthritic phenotype in mice receiving anti-DNAM-1 mAb at both clinical, ultrasound and histological assessments. But, we did not observe any difference between dnam1+/+ and dnam1−/− mice for incidence nor severity of clinical arthritis. Histological analysis revealed inflammatory scores similar in both groups, without evidence of erosion. Collagen antibodies levels were similar in all mice, confirming immunization with collagen.

**Conclusion:**

Despite some clues suggesting a role of DNAM-1 in arthritis, these complementary approaches demonstrate no contribution of *CD226*/DNAM-1 in the arthritic phenotype. These results contrast with previous studies showing a role *in vivo* of DNAM-1 in some autoimmune disorders.

**Electronic supplementary material:**

The online version of this article (doi:10.1186/s12950-015-0056-5) contains supplementary material, which is available to authorized users.

## Background

Rheumatoid arthritis (RA) is a frequent disease leading to joint destruction, deformity, and disability. Biologics have shown their efficacy in limiting joint destruction and have dramatically improved the outcome of RA-patients. However, some patients remain refractory or become nonresponder to these treatments, underlining the need in this context for new or complementary therapeutic strategies [1-3].

The pathogenesis of RA is characterized by chronic inflammation and synovial infiltration of immune cells [4,5]. It results from the combination of genetic susceptibility genes and environmental factors [6]. Until now, most of the susceptibility genetic factors identified are involved in inflammatory response and autoimmunity [7]. Furthermore, the majority of these genetic factors was also associated with other autoimmune diseases, which were not characterized by an arthritic phenotype, underlining the concept of a shared genetic background between autoimmune diseases [8-10].

Recently, a non-synonymous single nucleotide polymorphism (SNP) Gly307Ser (rs763361) in the *CD226* gene, which encodes the DNAX accessory molecule 1 (DNAM-1), has been associated with multiple autoimmune diseases including RA [11-18]. DNAM-1 is a 67 kDa type I membrane protein belonging to the immunoglobulin supergene family of receptors. It is constitutively expressed on the majority of CD4+ and CD8+ T cells, monocytes, natural killer cells, platelets and a subset of B cells. It is involved in the adhesion and co-stimulation of T cells in a Th1 pathway [19]. Interestingly, there are accumulating evidences suggesting a key role of T cells in the pathogenesis of RA characterized by a marked shift toward Th1 and Th17 phenotypes [20]. Furthermore, DNAM-1 was found to be significantly expressed on CD4+CD28- T cells from RA-patients and to be involved in co-stimulation of these cells [21]. Therefore, it remains to determine whether *CD226* Gly307Ser (rs763361) contributes specifically to the expression of the arthritic phenotype in RA or does it just reflect a common genetic background between autoimmune diseases. For this purpose, we aimed to validate *in vivo* this genetic susceptibility factor, using the collagen-induced arthritis (CIA) model, which is a widely used model for RA and has been important for understanding RA pathogenesis [22-24]. This might also reveal pathophysiological pathways leading to new potential therapeutic targets. We combined a targeted molecular approach with neutralizing anti-DNAM-1 monoclonal antibody (mAb) and a gene inactivation strategy using mice lacking DNAM-1 (dnam1−/−) in the CIA mouse model and demonstrated that inhibition of DNAM-1 does not have a direct influence on the development of inflammatory arthritis in mice.

## Methods

### Mice

DBA/1 mice were purchased from Janvier (Le Genest-St-Isle, France). Dnam1−/− mice have been described elsewhere [25]. C57/BL6 expressing DNAM-1 (dnam1+/+) were also purchased from Janvier (Le Genest-St-Isle, France). All mice were 6–8 weeks of age at the time of experimentation, were fed standard rodent chow and water ad libitum. To prevent from a cage effect, mice on different background or with different treatments were randomly assigned to each cage. The study was approved by the Cochin institute committee on animal care and its registered number is CEEA34.GC.052.12.

### Induction of CIA

For DBA/1 mice an emulsion was formed by dissolving 2 mg/ml bovine native collagen II (CII) (MD BioSciences, Zurich, Switzerland) overnight at 4°C in 10 mM acetic acid and combining it with an equal volume of complete Freund's adjuvant (CFA) emulsification (MD BioSciences, Zurich, Switzerland). DBA/1 mice were injected intradermally at three sites into the base of the tail with a total of 100 μl emulsion containing 200 μg CII emulsified in CFA. On day 21, an injection with CII in incomplete Freund’s adjuvant (IFA) was repeated as a booster [26]. CII solution and the emulsion with CFA or IFA were always freshly prepared. In this model, arthritis develops 20–30 days after the first collagen injection.

For C57/BL6 mice, the emulsion was formed by dissolving 4 mg/ml chicken CII overnight at 4°C in 10 mM acetic acid and combining it with an equal volume of CFA emulsification (MD BioSciences, Zurich, Switzerland). The booster was performed using the same protocol as for the priming immunization [27,28]. In this model, arthritis develops 50–60 days after the first collagen injection.

### Effect of DNAM-1 in CIA

To investigate whether prophylactic treatment with an anti-DNAM-1 neutralizing monoclonal antibody (mAb) might protect from the development of CIA, arthritis was induced in three groups of 7 DBA/1 mice (all males, 6–8 weeks old). In parallel, mice were treated intraperitoneally with the neutralizing anti-DNAM-1 mAb TX42 (rat IgG2a), at a concentration of 1.6 mg/ml, initially at a dose of 400 μg at day-1, then 200 μg every 5 days for 3 weeks, as previously described [29,30]. This antibody has been shown to inhibit DNAM-1 binding to its ligands CD155 and CD112 and inhibits activation of T cells in vitro [31]. Two control groups were treated for three weeks from day-1 with control rat IgG (MP Biomedicals) and PBS, respectively.

To determine whether DNAM-1 deficient mice were protected from CIA, arthritis was induced in 7 dnam1+/+ and 5 dnam1−/−. All dnam1+/+ and dnam1−/−mice were males of 6–8 weeks old.

### Evaluation of arthritis

#### Clinical assessment of arthritis

Mice were monitored for evidence of arthritis in their four paws using a blind procedure, by two examinators (G.C. and M.E.) and a clinical score based on disease severity was given for each mouse. Clinical assessment was performed two to three times per week, for up to 60 days in C57/BL6 mice and 35 days in DBA/1 mice as previously described [32]. Briefly, the date of disease onset was recorded, and the clinical severity of each joint or group of joints (toes, tarsus, ankle, wrist and fingers) was graded as follows: 0 (normal), 1 (swelling or focal redness of finger joints), 2 (mild swelling), 3 (severe swelling) or 4 (necrosis). Clinical scores of each joint (graded 0–4) were summed to yield the arthritic score and the severity of CIA was expressed as the mean score observed on a given day, as the mean of the score reached by mice during the experiment and as the mean of the maximal arthritic score reached by each mouse. The maximum score reached for each of the 10 joints was 4, so the maximum score of clinical arthritis reached for a single mouse on a given day was 40. Incidence of CIA was calculated by dividing the number of mice showing swelling of any paws (score > 1) with the number of total mice.

#### Ultrasound methodology

##### Animal preparation

Mice were sedated using 1.5% isofluorane in air (Minerve équipement vétérinaire, Esternay, France). They were attached on the heating pad in a supine position [33]. Hairs were removed from the knee and ankle using depilatory cream.

##### Evaluation of arthritic joints by ultrasound

This evaluation was performed in collaboration with the Small Animal Imaging Facility, which developed the ultrasound (US) examination in arthritic mice. Mice were monitored for evidence of arthritis in their knees, ankles, tarsus and toes by a blinded examinator (C.M), as previously described [34]. US examinations were performed using Ultrasound biomicroscope UBM (VEVO770, Visualsonics) either in B mode or in Doppler mode. A 60 MHz probe (RMV708) was used for optimal spatial resolution (with axial resolution up to 30 mm), and attached to an articulated arm (MP-Tec AG, Veltheim, Switzerland). The position was kept by tightening the articulated arm. Power Doppler imaging settings were defined as follows: the scan speed was set to 2 mm/s, the wall filter to 2.5 mm/s, the number of pulses to two radio-frequency cycles. Emitted power (ranging from 10 to 50%) and colour priority over grey scale were adjusted to allow visualisation of the vessels and remove the colour signal at the skin/tissue interface (Doppler artefacts). In B-mode images, each joint or group of joints was coted according to five different grades. Vascularisation was scored by the operator according to four different grades, depending upon the percentage of coloured pixels in the joint area, i.e. Grade 0: no detectable power Doppler signal; grade 1: one to two small colour Doppler areas (about 0.1 × 0.1 mm); grade 2: two to four medium colour Doppler areas (about 0.15 × 0.15 mm); grade 3: more than four large colour Doppler areas (over 0.2 × 0.2 mm).

On C57/BL6 mice, there was a power Doppler background level, which was not considered as pathological, since it was observed on naive C57/BL6 mice.

Six C57/BL6 and DBA/1 non-immunized mice (males of 6–8 weeks of age) were assessed as controls at B-mode and Doppler.

#### Histological assessment of arthritis

At the end of the experiment, i.e. day 35 in DBA/1 mice and day 62 in C57/BL6 mice, corresponding to the decrease in signs of arthritis, mice were killed by cervical dislocation, the paws of the mice were removed, fixed, decalcified and paraffin embedded. At least four serial sections were cut from each paw to ensure extensive evaluation of the arthritic joints. Sections (5 μm) were stained with hematoxylin and eosin and examined to search for inflammation, pannus formation, and cartilage and bone damage. All images were captured with a Nikon Eclipse 80i microscope (Nikon, Badhoevedorp, Netherlands) equipped with a DSP 3CCD camera (Sony, Tokyo, Japan). The histological severity of the arthritis was scored on a scale of 0–3 for synovitis (synovial proliferation, inflammatory cell infiltration) and erosions by two examinators (G.C. and M.E.), blinded to the diagnosis [35].

#### Assessment of DNAM1+T cells and T cell infiltrates

We aimed to quantify T cell infiltrates and DNAM1+T cells in arthritic joints from mice treated with anti-DNAM1, PBS or control IgG. We performed immunofluorescent staining in paws sections from 4 mice from each group (i.e. treated with anti-DNAM1, control IgG and PBS). Sections were deparaffinized, followed by antigen retrieval with Tris/EDTA/Tween, incubation with 5% bovine serum albumin in phosphate buffered saline for 1 hour to block non-specific binding. The number of T cells in the infiltrate was detected by staining for one hour at 4°C with polyclonal rabbit anti-mouse antibodies for CD3 (Abcam, Cambridge, UK). Alexa Fluor® 488 Goat Anti-rabbit antibodies (Life Technologies Saint Aubin, France) was used as secondary antibody for one hour at room temperature. For co-staining experiments, the expression of DNAM-1 and the number of T cells were detected by staining overnight at 4°C with polyclonal rabbit anti-mouse DNAM-1 antibody (Abcam, Cambridge, UK) and monoclonal rat anti-mouse antibodies for CD3 (Abcam, Cambridge, UK), respectively. Alexa Fluor® 488 Goat Anti-rat and Alexa Fluor® 594 Goat Anti-rabbit antibodies (Life Technologies Saint Aubin, France) were used as secondary antibody for one hour at room temperature. Nuclei were stained using DAPI. Slides were then mounted coverslip with a drop of mounting medium and stored in the dark at +4°C until analyze. All images were captured on a Zeiss Axio Observer Z1 microscope with a dry × 40 objective and CoolSNAP HQ2 CCD camera. Four mice were used for this experiment and 4 pictures were taken by mouse. The number of CD3+ and DNAM1+CD3+ cells were counted by two examinators blinded to the treatment.

#### Detection of anti-CII Ab by ELISA

Anti-CII antibodies were detected in the serum of mice. Mice were bled by cardiac puncture at the time of death, just after cervical dislocation. Sera were stored at – 20°C until use. ELISA assays were performed for the detection of Abs to CII by coating 96-well flat-bottom plates with 50 μl of CII (2 mg/ml in PBS) overnight at 4°C. The wells were then blocked by 2-h incubation at 4°C with 100 μl of PBS containing 2% (w/v) BSA. Next, 50 μl of serial twofold dilutions (beginning at 1/1600 dilution) of mouse sera in PBS containing 2% BSA and 0.1% Tween-20 were applied and incubated overnight at 4°C. Three washes in PBS/0.05% (v/v) Tween-20 were applied between all steps. Polyclonal rabbit anti-mouse labeled with horseradish peroxidase (HRP) (Dako, Glostrup, Danmark)(50 μl) diluted in PBS containing 2% BSA and 0.1% Tween-20 were next applied for 2 h at room temperature (dilution 1:200). Five washes in PBS/0.05% (v/v) Tween-20 were applied between all steps. Antibodies were detected by incubation with TMB (3,3', 5,5’-tetramethylbenzidine) (Sigma, Lyon, France) substrate for 45 minutes in the dark. Optical density was measured at 450 nm. A standard curve was created for each assay by including serial dilutions of a reference sample obtained from the pooled sera of all immunized mice on each plate. The reference sample was arbitrarily assigned an antibody concentration of 1 AU/mL. Antibody concentrations for each serum sample were obtained by reference to this standard curve and were expressed in relative titer to this reference sample. We also tested control sera of non-immunized mice to calculate a positivity threshold.

### Statistical analysis

All data analyses were performed using Graph Pad Prism 6.01 (2012). Data were presented as median (+/− IQR) for continuous variables and numbers (percentages) for categorical variables. The Mann–Whitney U test for non-related samples was used for statistical analysis. Correlation between US and clinical assessments for ankle, tarsus and toes was assessed using Spearman rank correlation coefficient (rho). A p-value of less than 0.05 was considered statistically significant.

## Results

### Anti-DNAM-1 mAb does not protect from the development of CIA

**Figure 1:**
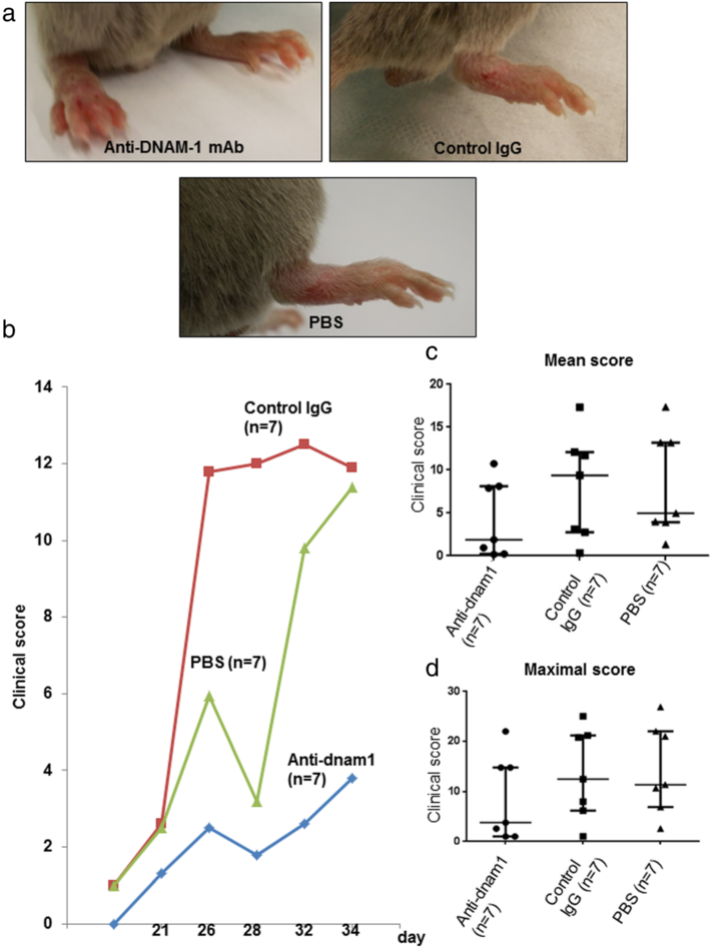
**Anti-DNAM-1 monoclonal antibody does not protect from the development of collagen-induced arthritis at clinical assessment. (a)** Clinical evidence of arthritis with redness and swelling of the paws involving tarsus, ankle and toes in the three groups: the first group was treated with anti-DNAM-1 monoclonal antibody (n = 7); the second was treated with control IgG antibody (n = 7) and the third with PBS (n = 7). Pictures were taken at day 30. **(b)** Clinical score similarly increased in the three groups from the injection to the end point. This score had a trend to be lower in the group injected with anti-DNAM-1 without significance. The blue curve represents the scores obtained in mice treated with AC anti-DNAM1 (n = 7), the red one those obtained in mice receiving control IgG (n = 7) and the green one the scores in mice treated with PBS (n = 7). **(c and d)** Median of the mean clinical score and of the maximal score were not significantly different between mice injected with anti-DNAM-1 and those treated with control IgG and PBS, but there was a trend for lower score in mice injected with anti-DNAM-1. Values are the median ± IQR.

The mouse model of CIA was used to evaluate the anti-arthritic potential of DNAM-1 inhibition *in vivo* using a neutralizing anti-DNAM-1 mAb. First evidence of arthritis appeared at day 19 post-immunization. Clinical evidence of arthritis (defined by at least one joint with a score > 1) was observed in 5/7 (71%) mice treated with anti-DNAM-1 mAb, 6/7 (86%) mice treated with control IgG and 7/7 (100%) mice injected with PBS (p = 0.51 and 0.13) (Figure 1a). Incidence of arthritis increased in the three groups during the time, but was less important in mice injected with anti-DNAM-1 mAb (Figure 1b). Median of the mean [± IQR] clinical score was 1.9 [0.2-8.1] in mice receiving anti-DNAM-1, 9.4 [2.8-12.1] in those treated with control IgG (p = 0.13) and 5.0 [3.9-13.2] in mice treated with PBS (p = 0.17) (Figure 1c).

Consistent with these previous results, the maximal score had a non-significant trend to be lower in mice receiving anti-DNAM-1 mAb (p = 0.37 and 0.25 versus mice injected with control IgG and PBS, respectively) (Figure 1d). The day of the maximal score was similar in the three groups (32nd day in the first two groups and 34st day in the latter; p = 0.67 and 0.78 versus control IgG and PBS, respectively) (Figure 1b).

**Figure 2:**
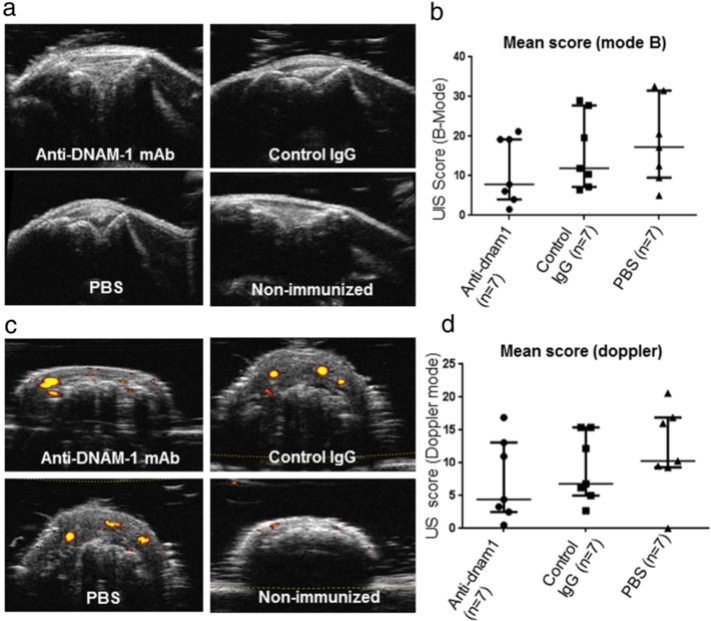
**Anti-DNAM-1 monoclonal antibody does not protect from the development of collagen-induced arthritis at US assessment. (a)** Examples of picture of the knees in B-mode assessment in mice injected with anti-DNAM1 (n = 7) (B-mode score = 0.5), those treated with control IgG antibody (n = 7) (B-mode score = 1.5) , those injected with PBS (n = 7) (B-mode score = 1.5) and control non-immunized mice (n = 6) (B-mode score = 0). **(b)** B-mode score was not significantly different between the three groups, despite a trend for lower scores in mice injected with anti-DNAM-1. **(c)** Examples of picture of inflammatory synovitis at Doppler assessment in tarsus of the three groups of mice (Doppler score = 1 in mice injected with anti-DNAM-1, whereas it was equal to 2 in the other groups). Example of Doppler assessment in tarsus of non-immunized mice (Doppler score = 0 in all 6 non-immunized mice). To note, there are artefacts signals Doppler on bone. **(d)** Doppler score was not significantly different between the three groups, despite a trend for lower scores in mice injected with anti-DNAM-1.Values are the median ± IQR.

To better evaluate the difference between the three groups, we combined an US approach, which allows an accurate assessment of both synovitis in B-mode and inflammatory activity with Doppler. US was consistent with our clinical assessment, as it revealed a non-significant trend for lower scores in B-mode assessment in mice treated with anti-DNAM-1(p = 0.25) (Figure 2a and b). Moreover, Doppler demonstrated a less inflammatory activity in mice receiving anti-DNAM-1 as compared to those treated with control IgG or PBS, but it did not reach significance (p = 0.43 and p = 0.41, respectively) (Figure 2c and d).

In all non-immunized mice, both B-mode and Doppler scores were equal to 0.

**Figure 3:**
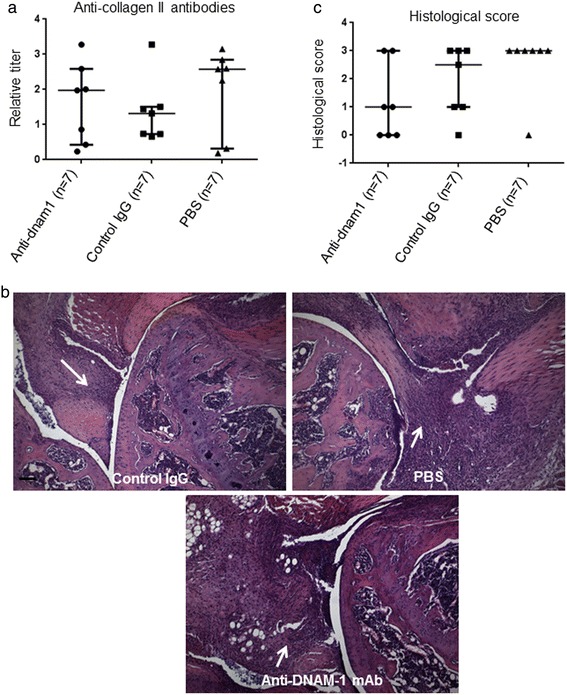
**Anti-DNAM-1 monoclonal antibody does not protect from the development of collagen-induced arthritis at histological assessment. (a)** Collagen antibodies levels detected by Elisa in mice injected with anti-DNAM-1 antibody, control IgG antibody and PBS. A standard curve was created for each assay by including serial dilutions of a reference sample obtained from the pooled sera of all immunized mice on each plate. The reference sample was arbitrarily assigned an antibody concentration of 1 AU/mL. Antibody concentrations for each serum sample were obtained by reference to this standard curve and were expressed in relative titer to this reference sample. Collagen antibodies levels are similar in the three groups, confirming the immunization with collagen. **(b)** Examples of knee sections stained by hematoxylin-eosin revealing inflammatory infiltrates (arrow) but no erosions in the three groups, i.e. treated with anti-DNAM-1 monoclonal antibody, treated with control IgG antibody and injected with PBS. Bar: 100μm. **(c)** Inflammatory score at histological assessment had a no significant trend to be lower in mice injected with anti-DNAM-1 as compared to those treated with control IgG and PBS. Values are the median ± IQR.

Collagen antibodies levels were similar in the three groups (p = 0.75 and p = 0.62, respectively), confirming the immunization with CII (Figure 3a).

Histological analysis revealed extensive inflammatory infiltrates in the synovium, without evidence of bone damage in all groups (Figure 3b). Inflammatory scores were not significantly different across the three groups, despite a trend for lower scores in those receiving anti-DNAM-1 (p = 0.35 and p = 0.10 versus mice injected with control IgG and PBS, respectively) (Figure 3c). To further assess the effect of anti-DNAM1 antibody, we quantified T cell infiltrates and DNAM1+T cells in each group of mice. We observed a significant reduction of the number of infiltrating T cells and the number of T cells expressing DNAM-in mice treated with anti-DNAM1 antibody as compared to mice injected with control IgG and PBS (Additional file 1: Figure S1).

DNAM-1 mAb was administered without serious adverse events for 3 weeks. No substantial changes were observed among mobility, activity, and comportment between mice treated with anti-DNAM-1 mAb or control IgG. Moreover, weight loss was <10% and no significant reduction of the food consumption was observed.

Although not significant, our results suggested a trend toward a less arthritic phenotype in mice treated with anti-DNAM-1 mAb. However, the molecular targeted strategy may be characterized by an incomplete blockade of the pathway targeted, unlike the gene inactivation strategy. Therefore to better assess the effect of invalidation of DNAM-1 in prevention of CIA, we decided to use mice deficient for DNAM-1, in which the pathway is completely blocked. We used these mice in a previous study and they were on a C57/BL6 background [29].

### Mice deficient for DNAM-1 are not protected from CIA

**Figure 4:**
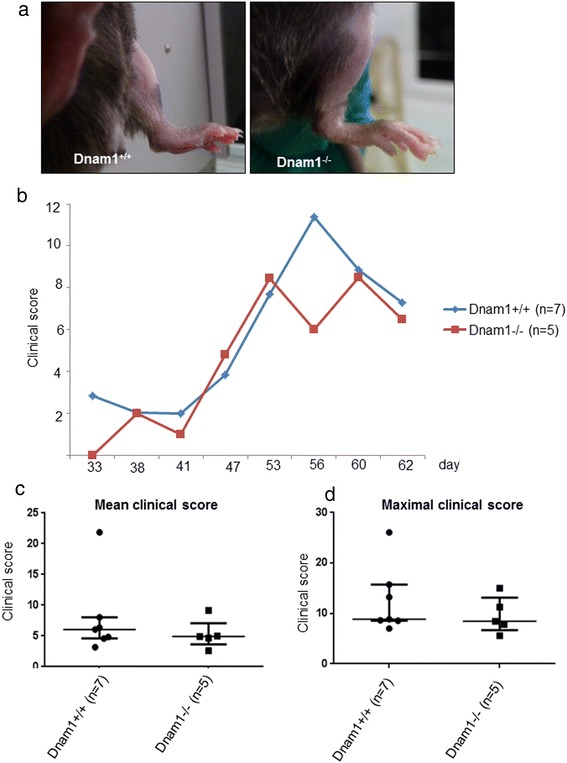
**Mice deficient for DNAM-1 are not protected from collagen-induced arthritis at clinical assessment. (a)** Examples of picture of clinical arthritis in paws from dnam1+/+ and dnam1−/− mice with redness and swelling of the paws involving tarsus, ankle and toes in both groups. Pictures were taken at day 54. 7 dnam1+/+ and 5 dnam1−/− mice were used. **(b)** Clinical score similarly increased in dnam1+/+ and dnam1−/− mice without significant difference. **(c)** Mean of the different clinical scores is not different between dnam1+/+ and dnam1−/− mice. **(d)** Maximal clinical score is similar in dnam1+/+ and dnam1−/− mice. Values are the median ± IQR.

To further evaluate the role of DNAM-1 in CIA, 5 dnam1−/− mice and 7 control dnam1+/+ mice were immunized with CII. First evidence of arthritis appeared at day 38. All, 7/7 (100%) dnam1+/+ and 4/5 (80%) dnam1−/− mice developed arthritis (p = 0.22) (Figure 4a). Incidence of arthritis increased similarly in both groups during the time (Figure 4b).Median of the mean [± IQR] clinical score was 6.0 [4.6-8.0] in dnam1+/+ mice versus 4.9 [3.6-7.1] in dnam1−/− mice (p = 0.60) (Figure 4c). Consistent with these results, maximal clinical score did not differ significantly between both groups (p = 0.33) (Figure 4d).The day of the maximal score was similar in both groups (53rd day in dnam1−/− mice and 56st day in dnam1+/+ mice, p = 0.78) (Figure 4b).

**Figure 5:**
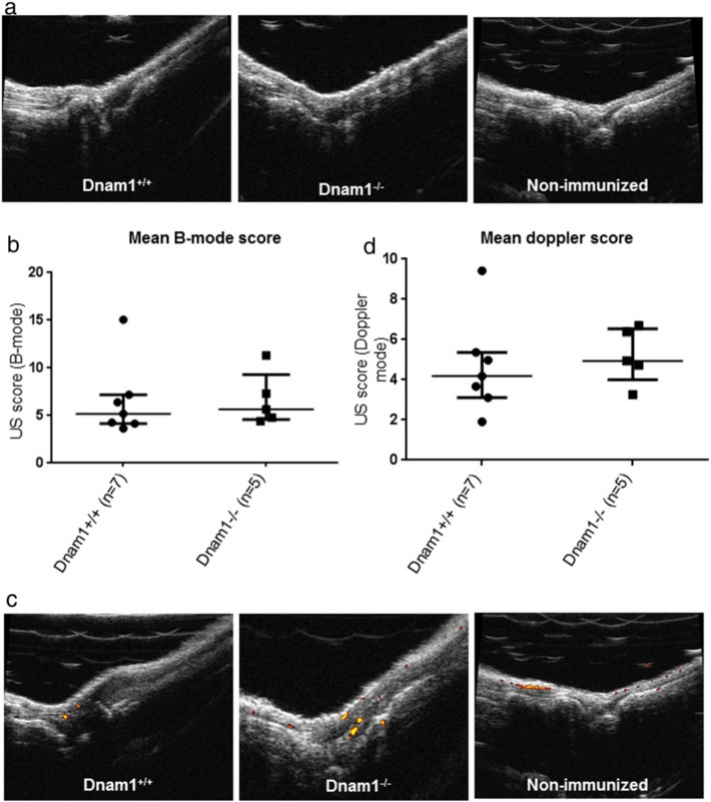
**Mice deficient for DNAM-1 are not protected from collagen-induced arthritis in B-mode. (a)** Examples of picture of ankle at ultrasound assessment in B-mode (B-mode score for the ankle = 1 in dnam1+/+ mice and 1.5 in dnam1−/− mice). Examples of ankle at B-mode assessment in control non-immunized mice (n = 6) (B-mode score = 0). **(b)** B-mode score was similar in dnam1+/+ and dnam1−/− mice. **(c)** Examples of picture of ankle at ultrasound assessment in Doppler (Doppler score for the ankle = 1 in dnam1+/+ mice and 1.5 in dnam1−/− mice). Example of Doppler assessment in ankle of non-immunized mice (Doppler score = 0 in all 6 non-immunized mice). To note, there are artefacts signals Doppler on bone. **(d)** Doppler score was not different between dnam1+/+ and dnam1−/− mice. Values are the median ± IQR.

We combined a B–mode US assessment to better evaluate arthritis in the mice and observed similar scores in dnam1+/+ mice (5.2 [4.2-7.2] and dnam1−/− mice (5.7 [4.6-9.3] in B-mode (p = 0.53) (Figure 5a and b). Of interest, Doppler, which is an accurate tool to measure inflammatory activity in cases of arthritis, did not demonstrate any difference between dnam1+/+ and dnam1−/− mice (scores: 4.2 [3.1-5.4] and 4.9 [4.0-6.5], respectively; p = 0.53) (Figure 5c and d). In all non-immunized mice, both B-mode and Doppler scores were equal to 0.

**Figure 6:**
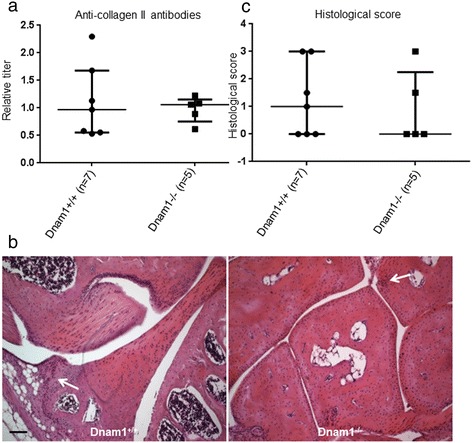
**Mice deficient for DNAM-1 are not protected from collagen-induced arthritis at histological assessment. (a)** Collagen antibodies levels detected by Elisa. A standard curve was created for each assay by including serial dilutions of a reference sample obtained from the pooled sera of all immunized mice on each plate. The reference sample was arbitrarily assigned an antibody concentration of 1 AU/mL. Antibody concentrations for each serum sample were obtained by reference to this standard curve and were expressed in relative titer to this reference sample. Collagen antibodies levels were similar in dnam1+/+ and dnam1−/− mice, confirming the immunization with collagen in all mice. **(b)** Example of a knee section stained by hematoxylin-eosin revealing inflammatory infiltrates (arrow) but no erosions in a dnam1+/+ mouse. Example of a tarsus section stained by hematoxylin-eosin revealing inflammatory infiltrates (arrow) but no erosions in a dnam1−/− mouse. Bar: 100μm. **(c)** Inflammatory score at histological assessment was not significantly different in dnam1+/+ mice and dnam1−/− mice. 7 dnam1+/+ mice and 5 dnam1−/− mice were used. Values are the median ± IQR.

Collagen antibodies levels were also similar in dnam1+/+ and dnam1−/− mice (p = 0.88) (Figure 6a).

Histological analysis revealed mild inflammatory infiltrates in the synovium, without evidence of bone damage (Figure 6b). Inflammatory scores were low and not significantly different in both groups (p = 0.79) (Figure 6c).

### Correlation between US and clinical assessments

We assessed correlation between US and clinical assessments in both experiments, for the joints, which were assessed by both methods (i.e. ankle, tarsus and toes) and observed a high correlation (rho: 0.71; p<0.01).

## Discussion

In the present study, we demonstrate that *CD226*/DNAM-1 may be not a relevant target in RA, using an *in vivo* approach in the CIA model. Despite several genetic studies revealing that *CD226* was a susceptibility genetic factor for RA in different ethnic groups [11-18], neither inactivation of DNAM-1 nor a molecular targeted strategy using a neutralizing mAb against DNAM-1 prevented from CIA. To assess rigorously the effect of invalidation of DNAM-1, we used four different validated parameters (clinical score, US score, histological assessment and collagen antibodies levels) [34]. All these four parameters displayed the same results, demonstrating no significant effect of invalidation or neutralization of DNAM-1 in CIA. This effect was not due to an arthrogenic effect of the antibody on its own, since we did not observe any clinical arthritis in non-immunized control mice treated with this mAb in a previous project [29].

Until now, genetic approaches have revealed that *CD226* Gly307Ser (rs763361) polymorphism was significantly associated with several different autoimmune diseases and the risk of multiple autoimmune diseases [11-18,36-38]. There is increasing evidence suggesting that autoimmune diseases share a genetic background and that autoimmune phenotypes represent pleiotropic outcomes of nonspecific disease genes [9,10,39]. Several susceptibility genes for autoimmune diseases have been identified without confirmation of their involvement in the development of the pathogenic phenotype. Therefore, it is crucial to proceed to their functional validation *in vivo*. So far, the contribution of *CD226*/DNAM-1 to the phenotype of several autoimmune diseases has been demonstrated *in vivo* in some murine models [29,38,40]. In a murine model of multiple sclerosis, i.e. experimental autoimmune encephalomyelitis, anti-DNAM-1 treatment delayed the onset and reduced the severity of the disease [40]. In a mouse model of acute graft-versus-host disease (GVHD), invalidation of DNAM-1 and an anti-DNAM-1 mAb were both associated with a milder GVHD and prolonged survival [38]. Recently, our group demonstrated that inhibition of DNAM-1 significantly ameliorated dermal fibrosis, in a murine model of systemic sclerosis [29]. In all these murine models, the blocking of DNAM-1 was associated with decreased infiltration of T cells, suggesting that it could reflect a general effect of DNAM-1 on inflammation rather that an effect linked to a specific genetic susceptibility for the autoimmune disease considered. Here we show that blocking *CD226*/DNAM-1 has no effect in another T cell-mediated inflammatory disease, despite a decrease in infiltrating T cells, consistent with what was observed in the mouse models of dermal fibrosis [29]. This suggests that other cells are involved in the physiopathology of CIA. This also highlights that DNAM-1 may be implicated in the development of a specific pathogenic phenotype, such as dermal fibrosis, encephalomyelitis and GVHD, but non RA.

In this report, we confirm that mice on a C57BL/6 background can develop clinical signs of arthritis with a high incidence around 100%, using a modified procedure. Incidence of arthritis in our study was much higher than in previous reports (60-70%) [27,28,41]. Several differences may account for this discrepancy: first we used a concentration of CII equal to 4 mg/ml instead of 2mg/ml, secondly we performed immunization only on male mice and thirdly male were younger than in previous studies. This slightly modified procedure (previously used by our team (unpublished results)) could increase the incidence of arthritis in C57/BL6 mice, but should be evaluated in further studies. This is of interest, since most of the knockout mice are on a C57/BL6 background. Nonetheless, the severity of clinical arthritis was lower in C57/BL6 mice than in DBA/1 mice, which is consistent with a previous study [27]. In this report, we also confirmed that arthritis develops later in mice on a C57/BL6 background (around 40th day) as compared to DBA/1 mice (on the 20th day) [27,28,41]. However, we observed a lower incidence and severity at histological assessment with low inflammatory scores in C57/BL6 mice than in DBA/1 mice.

US was proved to be a valuable method for evaluating arthritic lesions in mice on a DBA/1 background [34]. Here we confirm these results and suggest that US could also be used in other background, such as C57/BL6 with preliminary results, revealing high concordance with clinical analysis. Moreover, power Doppler allows visualizing and quantifying joint vascularization during the course of the disease. Therefore, the ability of US in investigating arthritic lesions in C57/BL6 mice should be evaluated in further studies.

Our study should be interpreted within its limitations. First, we could not exclude an immunization of our mice against this antibody. However, there were a significant reduction of infiltrating T cells, especially those expressing DNAM-1, following treatment with anti-DNAM-1 mAb suggesting that the Ab was biologically effective on these mice, despite no effect on the arthritic phenotype was detected. Moreover, we did not observe any bone and cartilage erosions unlike previous publications and induction of arthritis on the C57/BL6 background was challenging [27,28,41]. However, there was evidence of induction of arthritis with clinical signs of arthritis, inflammatory infiltrates at histology and production of CII antibodies. Nevertheless, we can suggest using other models, such as antigen-induced arthritis using mBSA or K/BxN serum-induced arthritis, to confirm our results.

In addition, we could not assess the correlation between clinical and US assessments for all joints, since some joints were assessed only clinically (i.e. wrists and fingers), whereas knees only were evaluated by US alone. However, our results demonstrate a high correlation between clinical and US evaluations for all joints investigated. Moreover, US is an operator-dependent method and here the assessment was only performed by one examinator. Nevertheless, this was performed by a trained investigator, who developed this strategy [34].

## Conclusion

Inhibition of DNAM-1, a critical new genetic factor in autoimmune disorders, does not prevent the development of CIA, despite its efficacy in other models of inflammatory diseases, such as bleomycin-induced fibrosis, encephalomyelitis and GVHD. This interesting result suggests that DNAM-1 might be involved in the development of a specific autoimmune phenotype and not in all inflammatory diseases. It remains to assess the efficacy of inhibition of DNAM-1 *in vivo* in other autoimmune diseases, in which *CD226* was identified as a susceptibility genetic factor, such as type 1 diabetes and systemic lupus erythematosus [11,13,36].

## Additional file

Additional file 1: Figure S1. Treatment with anti-DNAM-1 monoclonal antibody was associated with a decrease in infiltrating T cells and in T cells expressing DNAM-1 (a) Representative pictures showing decreased T cell infiltrates in mice injected with anti-DNAM-1 monoclonal antibody. T cells are identified by CD3 staining (in green), whereas nuclei are stained in blue. (b) Reduction of infiltrating T cells in the group of mice treated with the monoclonal antibody against anti-DNAM-1 (n = 4), compared to those treated with control IgG antibody (n = 4) and those injected with PBS (n = 4). (c).The number of infiltrating T cells expressing DNAM-1 was significantly lower in mice treated with the anti-DNAM1 monoclonal antibody (n = 4) as compared to those receiving either control IgG (n = 4) either PBS (n = 4). Values are the median ± IQR. **: p<0.01.

## Abbreviations

CII: collagen II
CIA: collagen induced-arthritis
CFA: complete Freund's adjuvant
DNAM-1: DNAX accessory molecule 1
GVHD: graft-versus-host disease
IFA: incomplete Freund's adjuvant
mAb: monoclonal antibody
RA: rheumatoid arthritis
SNP: single nucleotide polymorphism
US: ultrasound.
