# Supplementary figures and images for: Targeting CD226/DNAX accessory molecule-1 (DNAM-1) in collagen-induced arthritis mouse models
Source: J Inflamm (Lond). 2015 Feb 8;12:9. doi: 10.1186/s12950-015-0056-5 (PMC4327789; doi:10.1186/s12950-015-0056-5)

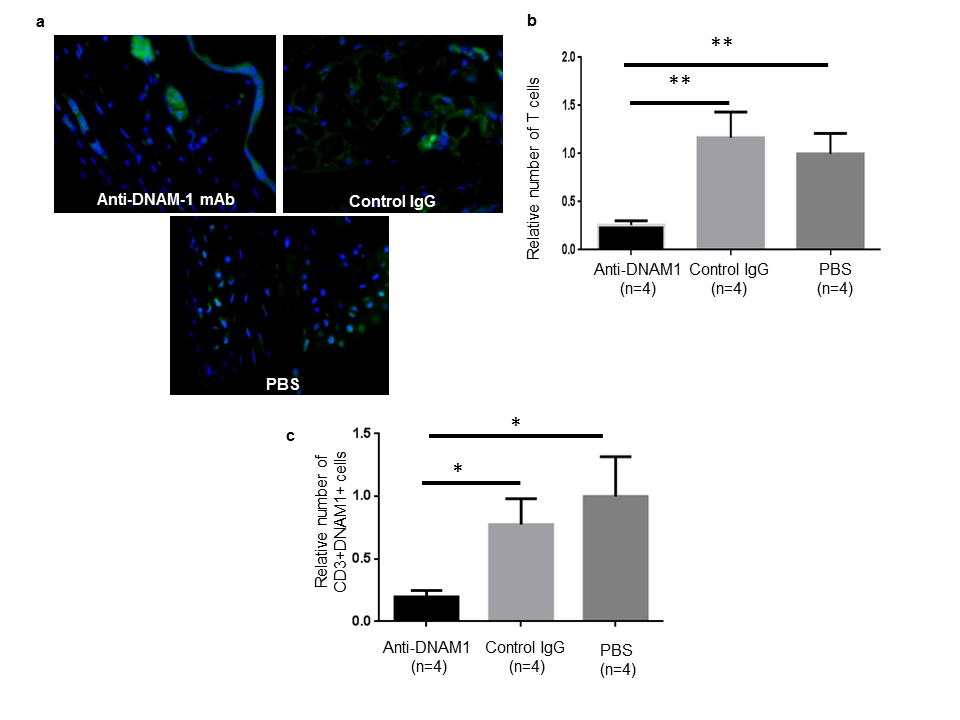

Supplement: Additional file 1: Figure S1. — Treatment with anti-DNAM-1 monoclonal antibody was associated with a decrease in infiltrating T cells and in T cells expressing DNAM-1 (a) Representative pictures showing decreased T cell infiltrates in mice injected with anti-DNAM-1 monoclonal antibody. T cells are identified by CD3 staining (in green), whereas nuclei are stained in blue. (b) Reduction of infiltrating T cells in the group of mice treated with the monoclonal antibody against anti-DNAM-1 (n = 4), compared to those treated with control IgG antibody (n = 4) and those injected with PBS (n = 4). (c).The number of infiltrating T cells expressing DNAM-1 was significantly lower in mice treated with the anti-DNAM1 monoclonal antibody (n = 4) as compared to those receiving either control IgG (n = 4) either PBS (n = 4). Values are the median ± IQR. **: p<0.01. [file 12950_2015_56_MOESM1_ESM.tiff]
